# Development and Validation of a Machine Learning Model to Predict Post-dispatch Cancellation of Physician-staffed Rapid Car

**DOI:** 10.14789/jmj.JMJ23-0031-OA

**Published:** 2024-05-10

**Authors:** TAKAAKI KAWASAKI, YOHEI HIRANO, YUTAKA KONDO, SHIGERU MATSUDA, KEN OKAMOTO

**Affiliations:** 1 Department of Emergency and Critical Care Medicine, Juntendo University Urayasu Hospital, Chiba, Japan; 1 Department of Emergency and Critical Care Medicine, Juntendo University Urayasu Hospital, Chiba, Japan

**Keywords:** machine learning, random forest, prediction, cancellation, rapid car

## Abstract

**Objectives:**

This study aimed to develop and validate a machine learning prediction model for post-dispatch cancellation of physician-staffed rapid car.

**Materials:**

Data were extracted from the physician-staffed rapid response car database at our Hospital between April 2017 and March 2019.

**Methods:**

After obtaining 2019 cases, we divided the dataset into a training set for developing the model and a test set for validation using stratified random sampling with an 8 : 2 allocation ratio. We selected random forest as the machine-learning classifier. The outcome was the post-dispatch cancellation of a rapid car. The model was trained using predictor variables, including 18 different reasons for rapid car request, age and gender of a patient, date (month), and distance from the hospital.

**Results:**

This machine learning model predicted the occurrence of post-dispatch cancellation of rapid cars with an accuracy of 75.5% [95% confidence interval (CI): 71.0-79.6], sensitivity of 81.5% (CI: 75.0-86.9), specificity of 70.8% (CI: 64.4-76.6), and an area under the receiver operating characteristic value of 0.83 (CI: 0.79-0.87). The important features were distance from the hospital to the scene, age, suspicion of non-witnessed cardiac arrest, farthest geographic area, and date (months).

**Conclusions:**

We developed a favorable machine learning model to predict post-dispatch cancellation of rapid cars in a local district. This study suggests the potential of machine-learning models in improving the efficiency of dispatching physicians outside hospitals.

## Introduction

The effectiveness of prehospital care provided by physicians remains controversial^1-4)^. Some papers have demonstrated the clinical benefits of physician-staffed rapid car compared to regular ambulances, such as shortened time to intravenous thrombolytic therapy for patients with ischemic stroke or improved survival in out-of-hospital cardiac arrest^5, 6)^. However, implementing physician-staffed emergency services require human and financial resources. Therefore, appropriate selection of cases for physician mobilization should be made.

Juntendo University Urayasu Hospital has rapid car since 2013. A rapid car is a car that has a physician on board to provide pre-hospital medical care. And Our rapid cars covers about 60km^2^. The crew members include one or two emergency physicians, one nurse, and two paramedics. The rapid car is operation from 9:00 am to 5:00 pm on weekdays and is dispatched about 700 cases in a year. They are requested by the fire department according to the criteria keywords included in the emergency request from the citizens. The criteria have 18 items including CPA, endogenous diseases and exogenous diseases, and allows over‐triage. After the dispatch, whether it was a cancelled or not, the reason for the dispatch, the time, and the location will be recorded.

The worst scenario for wasting medical resources is post-dispatch cancellation of physician-staffed emergency services. Post-dispatch cancellation of services is common due to the incomplete information provided by citizens and the delay in physician-staffed emergency services reaching the site. Although there are no clear criteria, ambulance crew might cancel a rapid car request when the ambulance have already arrived at the scene and was ready to transport to the hospital. They also might cancel it when they think that a patient does not need any care by a physician at the site. A rapid car crew sometimes cancel the request because they were working for another case. As cancelation of a rapid car does not involve any out-of-hospital activities by the physician and only results in wastage of human and financial resources, a strategy to reduce the number of post-dispatch cancellation is needed.

Few reports^7-10)^ have identified the factors responsible for post-dispatch cancellation. Understanding these factors is crucial as it would contribute to optimization of the dispatch criteria for prehospital emergency services. However, predicting the occurrence of cancellation before a physician-staffed vehicle is dispatched from the hospital would be more valuable.

The accuracy and effectiveness of prediction models using machine learning have been described in the recent years^11, 12)^. In addition to their high predictive performance, machine-learning models can return prediction results for each case. Thus, developing a prediction model based on machine learning may be a solution to minimize the number of post-dispatch cancellations.

Therefore, we aimed to develop and validate a machine-learning model to predict post-dispatch cancellation of physician-staffed rapid cars.

## Materials and Methods

### Data sources and Ethical considerations

The data for this study was obtained from the physician-staffed rapid response car (RRC) database at Juntendo University Urayasu Hospital between April 2017 and August 2020 The database included all rapid car cases dispatched from the hospital. This RRC system covered a total population of 660,435 and total area of 74.69 km^2^ in the districts of Urayasu and Ichikawa city in the Chiba prefecture of Japan. The following information were retrieved from the database: date; age, and sex of the patient; reason for RRC request; location of the scene; occurrence of cancellation; reason for cancellation; and the hospital to which the patient was transported. The details of the database and rapid car system at our institute have been described previously^10)^.

The study protocol was approved by the Ethics Committee of the Juntendo University Urayasu Hospital (E22-0450). The requirement for informed consent was waived owing to the retrospective observational design of the study. All the procedures conducted in this study were in accordance with the tenets of Declaration of Helsinki.

### Study population

**Figure 1:**
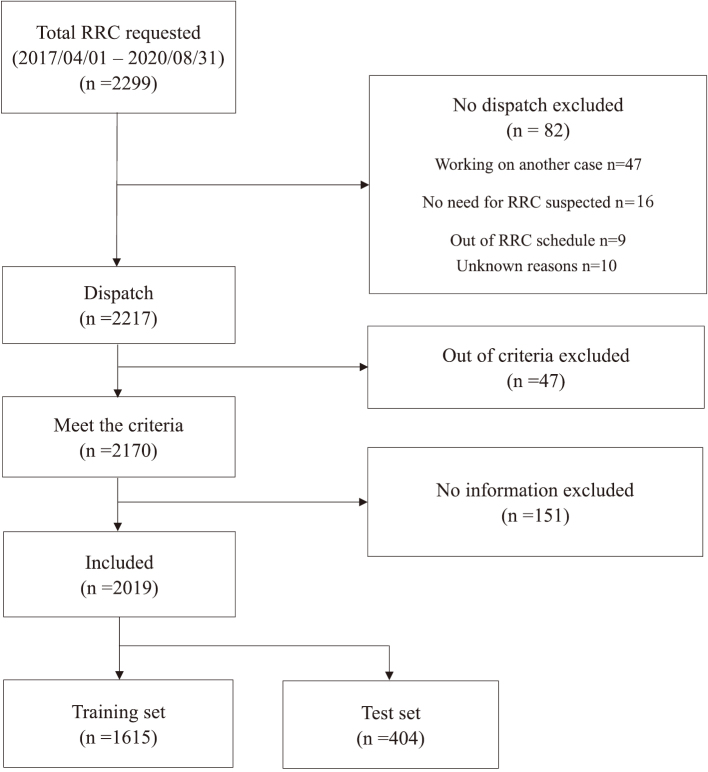
Flow diagram of this study RRC: rapid response car.

The study population comprised all the patients to whom RRC was dispatched from the hospital. A flow diagram of patient inclusion is shown in Figure 1. During the study period, 2299 requests were made for physician-staffed rapid car dispatch. However, 82 cases were excluded from analysis as the cars were not dispatched due to various reasons. Subsequently, we excluded 47 cases that did not meet the dispatch criteria and 151 cases with insufficient information. Thus, 2019 cases were included in this study. Finally, the dataset was divided into training (n=1615) and test sets (n=404) by stratified random sampling with an 8:2 allocation ratio.

### Predictor variables and outcome

Data regarding five variables, including date, age, sex, location of the scene, and reasons of requests, were retrieved from the database. We used these parameters as outcome predictors. Only the information of the month was used in the ‘date’ variable. To train the machine learning model, information on location of the scene was changed to distance between the hospital and the scene. In addition, the three kinds of categorical area (area 1: the nearest, area 3: the farthest district from the hospital) on the basis of the medical control region were delineated. The ‘reasons of request’ variable comprised 18 different reasons: suspicion of unwitnessed cardiac arrest, suspicion of witnessed cardiac arrest, chest pain and/or back pain, neurological deficit and/or severe headache, unconsciousness, convulsion, dyspnea, suspected hemorrhagic shock, traffic injury, fall, crush injury, penetrating injury, burn, drowning, airway obstruction, hanging, poisoning, and anaphylaxis. The outcome was the post-dispatch cancellation of a rapid car.

### Development and validation of a machine learning model

We trained and developed a random forest machine learning model using the training data for outcome prediction. During the development process, we performed 10-fold stratified cross-validation to avoid overfitting the model. In addition, the hyperparameters were optimized to obtain the best performance for outcome prediction. Internal validation of the developed model was performed using the test data. We computed the area under the receiver operating characteristic (AUROC) curve, sensitivity, specificity, positive likelihood ratio, negative likelihood ratio, positive predictive value, negative predictive value, and accuracy of the developed model for validation. In addition, important features for developing the model were assessed by calculating the normalized total reduction of the criterion brought about by the feature known as Gini importance^13)^.

Furthermore, machine learning prediction models for clinical use should be tuned according to the purpose of the model. Therefore, we compared the confusion matrices and statistical measures among six kinds of machine learning models with different binary classification thresholds ranging between 0.3 and 0.7 at intervals of 0.1.

### Statistical analysis and software library

Continuous variables, such as age and distance from the hospital, were reported as medians and interquartile range. Categorical variables were presented as raw counts and percentages. The Mann-Whitney U test was used to compare the median of the two samples. Chi-square test was used to compare frequencies. Two-sided significance level for all tests was set at 5% (p < 0.05). Analyses of the characteristics of the included cases were performed using EZR software version 3.3.2 (EasyR, Saitama Medical Center, Jichi Medical University, Saitama, Japan)^14)^. ScikitG-learn (version 0.21.3) with Python (version 3.7.4, in Anaconda 2019.10) was used for model development.

## Results

### Characteristics of study participants

The characteristics of the cohorts used for machine learning development and internal validation are presented in Table 1. The number of post-dispatch cancellations in 2019 cases were 891 (44.1%). Of these, males accounted for 58.7%, and the median age was 71 years old. The median distance from the hospital to the location of the scene was 7.8km. The most common reason for rapid car requests was unwitnessed, suspected cardiac arrest [522 cases, (25.9%)] followed by dyspnea [346 cases, (17.1%)]. Comparison between the training and test data showed no statistical difference in any of the variables used for the development of the machine learning algorithm, suggesting that they were evenly distributed.

**Table 1:** Characteristics of the datasets

Variable	All (n=2019)	Training data (n=1615)	Test data (n=404)	P value
Age (years)	71 [31]	71 [31]	72 [30]	0.61
Gender (male)	1185 (58.7%)	952 (58.9%)	233 (57.7%)	0.64
Distance from the hospital (km)	7.8 [9.5]	7.8 [9.5]	8.9 [10.7]	0.48
**EMS regional area**				0.44
Area 1	527 (26.1%)	419 (25.9%)	108 (26.7%)	
Area 2	478 (23.7%)	392 (24.3%)	86 (21.3%)	
Area 3	1014 (50.2%)	804 (49.8%)	210 (52.0%)	
**Month**				
March-May	434(21.5％)	336(20.8％)	98(24.2％)	0.10
June-August	579(28.6%)	473(29.2％)	106(26.2％)	
September-November	472(23.3%)	388(24.0％)	84(20.7％)	
December-February	534(26.4%)	418(25.8％)	116(28.7％)	
**Reason of RRC request**				0.59
Suspected cardiac arrest (unwitnessed)	522 (25.9%)	407 (25.2%)	115 (28.5%)	
Suspected cardiac arrest (witnessed)	63 (3.1%)	51 (3.2%)	12 (3.0%)	
Chest pain and/or back pain	180 (8.9%)	138 (8.5%)	42 (10.4%)	
Neurological deficit and/or severe headache	219 (10.8%)	182 (11.3%)	37 (9.2%)	
Unconsciousness	231 (11.4%)	183 (11.3%)	48 (11.9%)	
Convulsion	77 (3.8%)	64 (4.0%)	13 (3.2%)	
Dyspnea	346 (17.1%)	286 (17.7%)	60 (14.9%)	
Suspected hemorrhagic shock	29 (1.4%)	24 (1.5%)	5 (1.2%)	
Traffic injury	70 (3.5%)	54 (3.3%)	16 (4.0%)	
Fall	82 (4.1%)	68 (4.2%)	14 (3.5%)	
Crush injury	14 (0.7%)	8 (0.5%)	6 (1.5%)	
Penetrating injury	4 (0.2%)	3 (0.2%)	1 (0.2%)	
Burn	6 (0.3%)	5 (0.3%)	1 (0.2%)	
Drowing	12 (0.6%)	10 (0.6%)	2 (0.5%)	
Airway obstruction	39 (1.9%)	31 (1.9%)	8 (2.0%)	
Hanging	37 (1.8%)	34 (2.1%)	3 (0.7%)	
Poisoning	8 (0.4%)	6 (0.4%)	2 (0.5%)	
Anaphylaxis	56 (2.8%)	42 (2.6%)	14 (3.5%)	
**Outcome**				
Cancellation	891 (44.1%)	713 (44.1%)	178 (44.1%)	0.97

All categorical variables are shown as n (%). A continuous variables are shown as median [interquartile range]. EMS: Emergency Medical Service. RRC:Rapid Response Car

### Performance of the developed machine learning model

**Figure 2:**
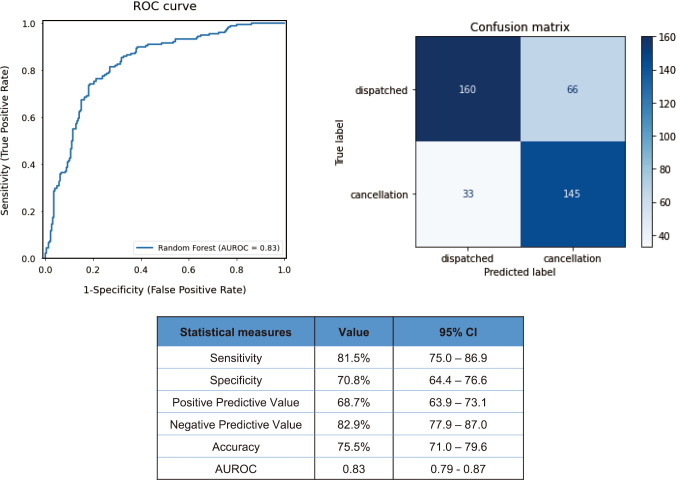
Performance of the developed machine learning model for outcome prediction in validation AUROC: the area under the receiver operating characteristic, CI: confidence interval.

The receiver operating characteristic (ROC) curve, confusion matrix, and statistical measures evaluated to validate the performance of the developed machine learning model in the test set are shown in Figure 2. The ROC curve and its AUROC value demonstrated good ability of the model to predict post-dispatch cancellation (AUROC: 0.83 [95% confidence interval (CI): 0.79-0.87]). The model predicted the outcome correctly with an accuracy of 75.5% (CI: 71.0- 79.6). The sensitivity, specificity, positive predictive value, and negative predictive value were 81.5% (CI: 75.0-86.9), 70.8% (CI: 64.4-76.6), 68.7% (CI: 63.9-73.1), and 82.9% (CI: 77.9-87.0), respectively.

### Variable importance for model development

**Figure 3:**
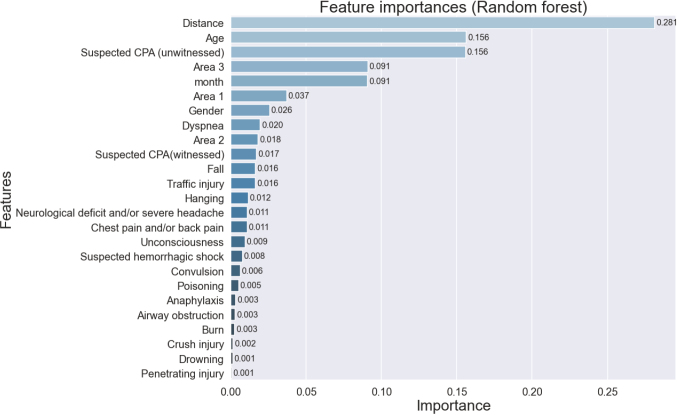
Feature importance to develop the machine learning model CPA: cardio-pulmonary arrest

Figure 3 shows the Gini importance of the variables used in the development of the model. Distance from the hospital was the most important feature in predicting the occurrence of post-dispatch cancellation, followed by patient’s age, unwitnessed/suspected cardiac arrest, farthest region from the medical control area (area 3), and the month of dispatch for the rapid car. These factors were among the top five important features.

### Comparison of statistical measures among machine learning models with different classification thresholds in validation

The statistical measures of the machine learning models with different binary classification thresholds are shown in Table 2. Under the threshold of 0.3, the developed model showed a sensitivity of 89.9% (CI: 84.5-93.9), specificity of 58.4% (CI: 51.7-64.9), positive predictive value of 63.0% (CI: 59.1-66.7), and negative predictive value of 88% (CI: 82.4-92.0). In contrast, the developed model under the threshold of 0.8 demonstrated a sensitivity of 38.2% (CI: 31.0-45.8), specificity of 91.2% (CI: 86.7-94.5), positive predictive value of 77.3% (CI: 68.3-84.3), and negative predictive value of 65.2% (CI: 62.3-67.9).

**Table 2:** Comparison of statistical measures among machine learning models with different classification thresholds in validation

Statistical measures	Threshold					
	0.3	0.4	0.5	0.6	0.7	0.8
Sensitivity	89.9(%)	86.0(%)	81.5(%)	76.4(%)	59.6(%)	38.2(%)
Specificity	58.4(%)	66.3(%)	70.8(%)	77.4(%)	85.8(%)	91.2(%)
Positive predictive value	63.0(%)	66.8(%)	68.7(%)	72.7(%)	76.8(%)	77.3(%)
Negative predictive value	88.0(%)	85.7(%)	82.9(%)	80.7(%)	72.9(%)	65.2(%)
Accuracy	72.3(%)	75.0(%)	75.5(%)	77.0(%)	74.2(%)	67.8(%)

AUROC: the area under the receiver operating characteristic, CI: confidence interval.

## Discussion

To the best of our knowledge, this study is the first to develop and validate a machine learning model to predict the occurrence of post-dispatch cancellation of physician-staffed emergency service. Using a single-center database, we developed a random forest-based prediction model that showed favorable performance in predicting post-dispatch cancellation in the internal validation with a reasonably high AUROC of 0.83.

Several studies have demonstrated the effect of prehospital care^15, 16)^. However, only a few studies have focused on human or financial cost-effectiveness of prehospital care^7-9)^. Although there have been few reports on cost-effectiveness of prehospital care using helicopters, physician-staffed RRC has received little attention.

It is important that overtriage of RRC cancellations should be tolerated because it is hard to select the exact patients who receive benefits by a rapid intervention of a physician at the site. However, we suggest that post-dispatch cancellation should be debated more seriously as it meant that nurses and physicians acted ineffectively outside the hospital. Rapid car stuff including physicians and nurses not only care the patients in the prehospital settings, but they also care other patients in the hospital. We believe that their presence in hospitals may have elevated the efficiency of in-hospital patient care of other sick patients. In such scenario, it is important to optimize the distribution of valuable human resources.

As the rapid car system at our institute has a very low dispatch threshold, it has a very high post-dispatch cancellation rate (44.1%). Thus, the database with balanced outcomes used in this study was valuable for assessing the optimization of post-dispatch cancellation rate by creating and validating machine learning models. Our machine learning model showed favorable predictive performance for post-dispatch cancellation, with an AUROC of 0.83. Thus, we might have a chance to optimize the rate of post-dispatch cancellation. In other words, we could decide not to dispatch to the site in some cases by using the predictive model when the RRC was requested. It is sure that the final decision in the medical situation should be done by medical professionals, however; the data-driven prediction such as machine learning would support us make important decisions^17-19)^. The prediction model is especially useful for medical professionals when human prediction is difficult.

Nevertheless, maintaining a fine balance between therapeutic effectiveness and resource consumption remains challenging. Reduction in the cancellation rate using the prediction model would be accompanied by an increased number of cases of no RRC dispatch wherein RRC involvement would have resulted in beneficial effects on patient outcome. The acceptable cancellation rate for RRC should change depending on the circumstance of the region or sufficiency of human resources of the facility. From this perspective, we developed a variety of machine learning prediction models by altering the binary threshold of positivity of prediction. As shown in Table 2, alteration of the binary thresholds provided a choice of prediction models with a variety of sensitivity and specificity. In contrast to the existing and conventional prediction tools, machine learning models can be tuned specifically. Therefore, medical facilities can select the best model to fit their purpose and achieve their acceptable level of RRC cancellation rate.

The most critical feature for the development of our machine learning model was the distance between the patient location and the hospital. If the distance from the scene to the hospital where the RRC is dispatched is long, it is more likely that the first arriving emergency medical service (ambulance crew) will decide where to transport the patient before the RRC arrives, hence the cancellation rate of the RRC might increases.

Interestingly, patient age and month of request for RRC were also key predictors in developing the model. As whether to accept patient transport to the hospital is usually dependent on the decision of each hospital’s physician in Japan, it is possible that patient age or seasonal increase in patient transport may have affected the acceptance rate of hospital transport and thus, led to the alteration of cancellation rate of RRC. For example, elderly patients with a variety of comorbidity or shortage of hospital beds in winter might be strong negative factors for the determination of the hospital where the patient will be transported. In this case, the need for RRC may increase and the cancellation rate would decrease.

Assessment of the feature importance of the model also provides us information to modify the conventional request criteria for RRC from the fire department. In our cases, alteration of the covering area of the RRC or removing unwitnessed/suspected cardiac arrest from the reason of RRC request is the candidate for new rule for RRC dispatch to decrease the cancelation rate, although we should consider the negative clinical effects due to these changes.

This study had a few limitations. First, our machine learning model was developed using a single-center database. Therefore, the results cannot be generalized to other institutions or regions. Development and validation of a machine learning model using datasets derived from each facility is anticipated. Second, very few important features, such as crush injury, drowning, and penetrating injuries, were used to develop the model. However, it is important to exercise caution when interpreting the impact of these parameters on post-dispatch cancellation owing to the limited sample size of the data. Third, sometimes it is difficult to obtain all the necessary information for designing a prediction model. As it is required that an early decision should be made for dispatching RRC, we should not stick to obtaining sufficient prehospital information to use the prediction model. Finally, the machine learning model requires the input of variables in the application when used in clinical settings. Thus, its usability for physicians might be inferior to the conventional RRC dispatch criteria. However, this disadvantage can be overcome by using technologies such as speech recognition.

In conclusion, we developed a favorable machine learning model to predict post-dispatch cancellation of rapid cars in a local district. Although each medical institution would have to adjust the model according to the region or facility, the current study demonstrated the potential of the machine learning-based prediction model to optimize post-dispatch cancellation of physician-staffed rapid cars.

## Funding

No funding was received.

## Author contributions

TK and YH analyzed and interpreted the patient data regarding post-dispatch cancellation of physician-staffed rapid response car. All authors have read and approved the final manuscript.

## Conflicts of interest statement

YH is the Chief Executive Officer, MedPop Co. Ltd. None of the other authors declare no conflict of interest.
